# Overexpression of Pygopus-2 is required for canonical Wnt activation in human lung cancer

**DOI:** 10.3892/ol.2013.1691

**Published:** 2013-11-19

**Authors:** SHI-YONG ZHOU, MEI-LIN XU, SHAO-QING WANG, FANG ZHANG, LEI WANG, HUA-QING WANG

**Affiliations:** 1 Department of Medical Oncology, Tianjin Medical University Cancer Institute and Hospital, Tianjin 300060, P.R. China; 2 Department of Pathology, Tianjin Chest Hospital, Tianjin 300051, P.R. China; 3 Longyao Hospital, Longyao County, Xingtai, Hebei 055350, P.R. China; 4 Tsinghua University Graduate School at Shenzhen, Division of Life and Health Sciences, Shenzhen, Guangdong 518055, P.R. China; 5 Department of Thoracic Surgery, Fourth Hospital of Hebei Medical University, Shijiazhuang, Hebei 050011, P.R. China

**Keywords:** Pygopus-2, Wnt activation, lung cancer, RNAi, inhibition

## Abstract

Lung cancer is the most common cause of cancer-related mortality worldwide. It is necessary to improve the understanding of the molecular mechanisms involved in lung cancer in order to develop more effective therapeutics for the treatment of this fatal disease. The canonical Wnt signaling pathway has been known to be important in a number of cancer types, including lung cancer. Pygopus (Pygo) is a recently identified downstream component of the Wnt signaling pathway required for β-catenin/T-cell factor (TCF)-dependent transcription. However, the role of Pygo in lung cancer remains to be elucidated. The present study showed that Pygo2 is overexpressed in human lung cancer tissue samples and cell lines. Expression levels of Pygo2 were found to correlate with cytosolic β-catenin protein levels in the samples examined. Co-immunofluorescent staining showed that Pygo2 protein accumulated in the nuclei and colocalized with nuclear β-catenin in lung cancer cell lines expressing Pygo2. To investigate the functional importance of the Pygo2 overexpression in lung cancer, short hairpin RNA (shRNA) was used to knockdown Pygo2 mRNA in lung cancer cells expressing the gene. Pygo2 shRNA was observed to inhibit cell proliferation and decrease β-catenin/TCF-dependent transcriptional activity *in vitro*. Furthermore, Pygo2 shRNA significantly suppressed lung cancer xenograft models *in vivo* (P<0.05). These results suggested that Pygo2 is a putative therapeutic target for human lung cancer and overexpression of Pygo2 may be important for aberrant Wnt activation in lung cancer.

## Introduction

Lung cancer is the most common type of cancer and the most frequent cause of cancer-related mortality worldwide. Non-small cell lung cancer (NSCLC) is the most common type and represents ~80–85% of all types of lung cancer (1). Current treatment strategies for lung cancer include surgical resection, chemotherapy, radiation therapy, targeted therapy or a combination of treatments depending on the type of lung cancer and its stage level. Despite advances made in these treatments, lung cancer remains highly lethal with a five-year survival rate of <15% (2). Therefore, new effective therapies for lung cancer are urgently required. An increased understanding of the molecular mechanisms underlying lung cancer development and progression are likely to lead to the design of improved targeted therapies in the treatment of this fatal disease.

Mammalian Wnt proteins comprise a family of 19 highly conserved and secreted glycoproteins. Secreted Wnt ligands have been shown to activate signal transduction pathways and trigger changes in gene expression, cell behavior adhesion and polarity (3). Receptors for the Wnt proteins are the Frizzled (Fzd) family of receptors. Transduction of Wnt signaling begins with Wnt ligands binding to the cysteine-rich domain of the Fzd receptors at the cell membrane initiating the ‘canonical’ or ‘non-canonical’ pathway (3). In the canonical Wnt pathway, Wnt binds to the Fzd receptors, activates Dishevelled (Dvl) and disassembles the β-catenin ‘destruction complex’, which prevents the phosphorylation and subsequent ubiquitination of β-catenin, resulting in β-catenin stabilization and accumulation in the cytoplasm. Stabilized β-catenin enters the nucleus, where it complexes with T-cell factor (TCF)/lymphoid enhancer-binding factor 1 transcription factors, B-cell lymphoma-9 (Bcl-9) and Pygopus (Pygo) to regulate the transcription of downstream target genes (3). The essential role of Pygo in canonical Wnt signal transduction has been mainly studied in *Drosophila* development. Its conserved C-terminal plant homeodomain has been shown to be required for association with Bcl-9, an adaptor protein that directly binds β-catenin and targets it to the nucleus. Once tethered to β-catenin/TCF, the N-terminal homology domain of Pygo has been proposed to activate target gene expression (4,5).

Aberrant activation of the canonical Wnt signaling pathway is associated with a variety of human cancers, including thoracic malignancies. For example, Wnt-1 and -2 are upregulated in NSCLC (6,7). Coexpression of Wnt-7a and Fzd-9 has been shown to inhibit the cell growth of NCSLC cell lines (8). Dvl is overexpressed in 75% of microdissected NSCLC tissues (9). In addition, methylation silencing of secreted Wnt antagonists, Wnt inhibitory factor-1 and secreted Fzd-related proteins, has been previously reported to be associated with aberrant Wnt activation in lung cancer (10–12). Mutations in key Wnt signaling genes, such as adenomatous polyposis coli or β-catenin, frequently found to correlate with colon cancer, appear to be rare in lung cancer (3). Thus, the Wnt pathway may be activated upstream of β-catenin (4,5,13). While a few previous studies have suggested that Pygo family members may be involved in β-catenin/TCF driven transcription in colorectal and breast cancer cells (5,13), the role that Pygo proteins may play in lung cancer, however, remains to be elucidated. The current study sought to investigate whether Pygo2 is important in aberrant activation of the Wnt signaling pathway in human lung cancer. Pygo2 expression in fresh human lung cancer tissue specimens and cell lines was examined, as well as the correlation between Pygo2 function and the canonical Wnt pathway in lung cancer cells.

## Materials and methods

### Cell lines and tissue samples

NSCLC cell lines were obtained from the China Center for Type Culture Collection (Wuhan, China) and cultured in RPMI 1640 medium. All cell cultures were supplemented with 10% fetal bovine serum (Invitrogen Life Technologies, Carlsbad, CA, USA), penicillin (100 IU/ml) and streptomycin (100 μg/ml) (Invitrogen Life Technologies). Cells were then cultured at 37°C in a humid incubator with 5% CO_2_.

Fresh lung cancer and adjacent normal lung tissues from patients were collected at the time of surgical resection and immediately snap-frozen in liquid nitrogen at theTianjin Medical University Cancer Institute and Hospital and Tianjin Chest Hospital (Tianjin, China). These tissue samples were kept at −80°C prior to use. Written informed consent was obtained from the patient and the study was approved by the ethics committee of Tianjin Medical University Cancer Institute and Hospital (Tianjin, China).

### RNA extraction and semi-quantitative reverse transcription-polymerase chain reaction (RT-PCR)

Total RNA was extracted from lung cancer cell lines and tissues using the TRIzol reagent [Tiangen Biotech (Beijing) Co., Ltd., Beijing, China] according to the manufacturer’s instructions. Semi-quantitative RT-PCR was performed as follows: cDNA was produced using avian myeloblastosis virus reverse transcriptase (Promega Corporation, Madison, WI, USA) and N9 random primers; and PCR was performed in GeneAmp 2700 (Applied Biosystems, Carlsbad, CA, USA) using the cDNA as template. Taq enzyme and PCR reagents were purchased from Tiangen Biotech (Beijing) Co., Ltd. and primers were purchased from Sangon Biotech (Shanghai) Co., Ltd. (Shanghai, China). The following primer sequences were used for human Pygo2: Forward, 5′-GCATCCAACCCTTTTGAAGATGAC-3′; and reverse, 5′-TCAGCCAGGGGGTGCCAAGCTGTTG-3′. The housekeeping gene, β-actin, was amplified as an internal control. The following PCR conditions were used: 94°C for 15 sec, 55°C for 30 sec and 72°C for 30 sec for 35 cycles, followed by a final extension at 72°C for 10 min. Semi-quantitative RT-PCR products were analyzed on 1% agarose gel electrophoresis and stained with ethidium bromide.

### Western blotting and immunofluorescent staining

Cytosolic proteins were prepared as follows: Cell pellet was suspended in hypotonic buffer [2 mM Tris-Cl (pH 7.5), 25 mM NaF and 1 mM EDTA), set on ice for 30 min and then spun down at 235,000 × g in an ultracentrifuge (Optima Max; Beckman Coulter, Miami, FL, USA) at 4°C for 30 min. The supernatant was then collected as cytosolic proteins. Total protein extraction was performed using M-PER Mammalian Protein Extraction Solution (Thermo Fisher Scientific, Waltham, MA, USA). Proteins were separated on 4–15% gradient SDS-polyacrylamide gels and transferred onto Immobilon-P membranes (Millipore, Billerica, MA, USA). Immunofluorescent staining was performed following standard procedure. Primary antibodies used were anti-Pygo2 (1:200; Santa Cruz Biotechnology, Inc., Santa Cruz, CA, USA), anti-β-actin (1:5,000; Sigma-Aldrich, St. Louis, MO, USA) and anti-β-catenin (1:2,000; Sigma-Aldrich). Antigen-antibody complexes were detected by an enhanced chemiluminescence blotting analysis system (Amersham Pharmacia Biotech, Amersham, UK).

### Transfection and RNA interference

Control (non-silencing) and the two Pygo2 short hairpin RNAs (shRNAs; all in pRFP-C-RS vector) were purchased from OriGene (Beijing, China). The following primers targeting human Pygo2 sequences were used: shRNA-1, 5′-CCTGCATACTCACAT CTGACGGAGTTTGC-3′; and shRNA-2, 5′-CTCTGCCTC AAGACCAAGGAGATCCAGTC-3′. Cell lines were plated in six-well plates with fresh media without antibiotics for 24 h prior to transfection. Transfection was performed using Lipofectamine 2000 (Invitrogen Life Technologies) according to the manufacturer’s instructions. Transfected cells were replated in 10-cm dishes for selection with G418 (500 μg/ml; Invitrogen Life Technologies). Stable transfectants were maintained in regular medium with G418 (300 μg/ml) for further analysis.

### Cell proliferation assay

The cell growth rate was determined by the CellTiter 96 Aqueous Non-Radioactive Cell Proliferation Assay kit (Promega Corporation). The stable cell lines were plated into 96-well tissue culture plates with a number of 5×10^2^ cells/well. The MTS solutions were added to the medium at various time points and incubated for 1.5 h. The absorbance at 490 nm was measured using a microplate reader (model 680; Bio-Rad, Hercules, CA, USA).

### Colony formation assay

In total, 500 individual cells of the stable lines were seeded in 10-cm dishes and cultured for two weeks. Colonies were then fixed by 10% formalin (Thermo Fisher Scientific), stained with 0.5% crystal violet (Thermo Fisher Scientific) and counted.

### Xenograft model

The mice experiments were conducted in the animal facility of the Tianjin Medical University Cancer Institute (Tianjin, China) and approved by the Institutional Animal Care and Use Committee. Lung cancer xenografts were established with 6-week-old female BALB/c nude mice. Briefly, A549 and H1299 cells stably transfected with control or Pygo2 shRNA were trypisnized and resuspended in phosphate-buffered saline (pH 7.4). The cell suspensions were then mixed with Matrigel (vigorous; volume ratio, 1:1) at 4°C. The mixture containing 5×10^6^ cells in a volume of 100 μl was s.c injected into the flanks of female mice (five mice/group). Tumor volume was determined by the following formula: V = 0.5(L × W^2^), where L is length and W is width.

### Statistical analysis

Statistical analysis was performed using GraphPad Prism 5.0 for Windows (GraphPad Software, Inc., La Jolla, CA, USA). Data are presented as the means ± standard deviations (error bars) of three independent experiments performed in triplicate. The difference between groups was determined by Student’s t-test and P≤0.05 was considered to indicate a statistically significant difference.

## Results

### Overexpression of Pygo2 in primary lung cancer tissue samples and cell lines

**Figure 1:**
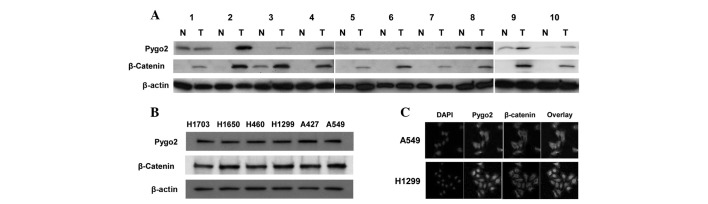
Western blot analysis of (A) freshly resected human lung cancer samples and their matched normal lung controls, and (B) human lung cancer cell lines. β-actin was used as a loading control. (C) Immunofluorescent staining of Pygo2 and β-catenin in human lung cancer cell lines, A549 and H1299. Pygo2, Pygopus-2; T, tumor; N, normal.

Western blot analysis was first used to examine the expression of Pygo2 and cytosolic β-catenin proteins in human primary lung cancer tissue samples (Fig. 1A). In the ten tissue samples from lung cancer patients examined, upregulation of Pygo2 expression was observed in 90% (9 out of 10) of the tumor samples when compared with their matched normal lung tissues. In one case, the Pygo2 expression was detected in the cancerous and matched normal tissue samples. Notably, the protein levels of Pygo2 were found to correlate with those of cytosolic β-catenin in the lung cancer tissue samples examined. Western blot analysis was also used to examine and compare the expression of Pygo2 and cytosolic β-catenin proteins in human lung cancer cell lines (Fig. 1B). Pygo2 and cytosolic β-catenin proteins were correlatively expressed in the lung cancer cell lines (A549, A427, H1299, H460, H1650 and H1703) that were examined, which was consistent with the observation in lung cancer tissue samples. In addition, co-immunofluorescent staining of Pygo2 and β-catenin proteins was performed and confirmed that Pygo2 protein accumulated in the nuclei and colocalized with nuclear β-catenin in lung cancer cell lines (Fig. 1C). Since aberrant overexpression of Wnt ligands has been previously reported in human lung cancer (6,14), the results of the present study suggested that there may be a functional significance of the Pygo2 overexpression in aberrant activation of Wnt signaling in human lung cancer.

### shRNA knockdown of Pygo2 suppresses the canonical Wnt pathway in lung cancer cells

**Figure 2:**
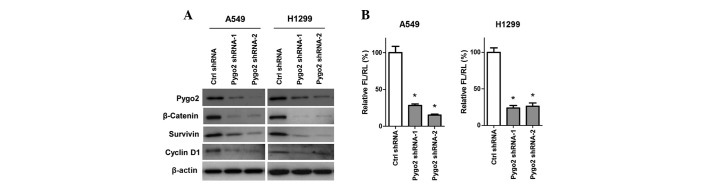
Pygo2 shRNAs suppress the canonical Wnt pathway in lung cancer cells *in vitro*. (A) Two independent Pygo2 shRNAs (shRNA-1 and -2) suppressed the canonical Wnt pathway in human lung cancer cell lines, A549 and H1299. Western blot analysis was used to examine protein expression in these cell lines stably transfected with control or Pygo2 shRNA. Actin was used as a loading control. (B) TOP/FOP assay in lung cancer cell lines stably transfected with control or Pygo2 shRNAs. Experiments were performed in triplicate and data are presented as the mean ± SD (error bars). Pygo2, Pygopus-2; shRNA, short hairpin RNA; Ctrl, control; FLRL, Firefly luciferase/Renilla luciferase.

To investigate the function of Pygo2 in human lung cancer, the effects of inhibiting Pygo2 expression in the canonical Wnt signaling pathway were examined in lung cancer cells. In total, two Pygo2-targeted shRNAs with independent sequences were used to silence Pygo2 mRNA expression in all experiments to avoid possible off-target effects produced by shRNA (15). First it was confirmed that the stably transfected Pygo2 shRNAs inhibited the Pygo2 expression in lung cancer cell lines (A549 and H1299) expressing the gene, whereas the non-silencing control shRNA exhibited no effect (Fig. 2). The cytosolic level of β-catenin protein and β-catenin/TCF-dependent transcriptional activity were then analyzed by TOP/FOP luciferase reporter assay, respectively. These two assays provide important signatures of the canonical Wnt activation (6,14), to explore the effect of Pygo2 shRNA on Wnt/β-catenin signal transduction. Cytosolic β-catenin protein levels and β-catenin/TCF-dependent transcriptional activity were found to be downregulated following the treatment with the two Pygo2 shRNAs in the lung cancer cell lines examined (Fig. 2A and B), indicating that Pygo2 functions as a positive regulator of the canonical Wnt/β-catenin signaling pathway in these cells. To further confirm the suppression of the Wnt pathway by inhibition of Pygo2 expression, the transcription of specific downstream target genes of the Wnt/β-catenin pathway, such as cyclin D1 and survivin (6,14), was examined in the lung cancer cells stably transfected with the Pygo2 shRNAs. The protein expression of cyclin D1 and survivin was found to be downregulated following Pygo2 knockdown (Fig. 2A). Overall, these results suggested that Pygo2 may be functionally important for β-catenin/TCF-dependent transcriptional activity and aberrant activation of the canonical Wnt signaling pathway in human lung cancer cells.

### shRNA knockdown of Pygo2 inhibits the proliferation of lung cancer cells in vitro

**Figure 3:**
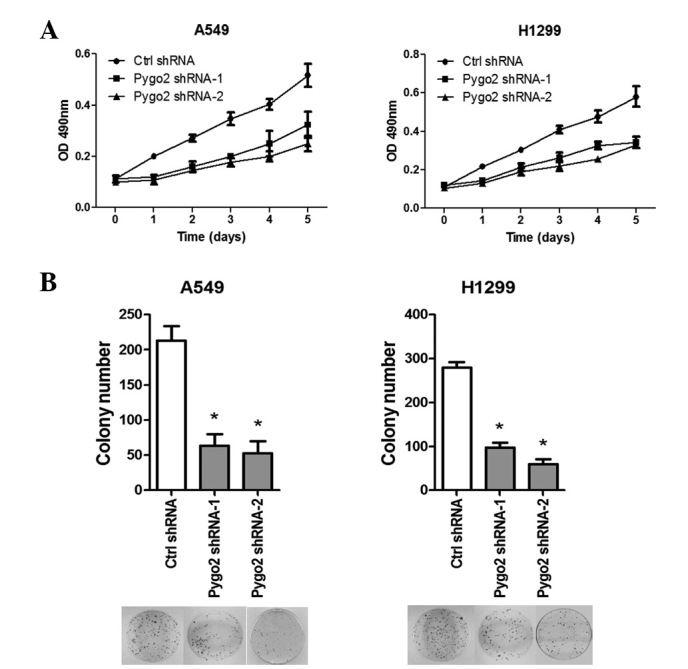
Pygo2 shRNAs suppress lung cancer cell proliferation *in vitro*. (A) MTS and (B) colony formation assays for A549 and H1299 stably transfected with control or Pygo2 shRNA. Experiments were performed in triplicate and data are presented as the mean ± SD (error bars). Pygo2, Pygopus-2; shRNA, short hairpin RNA; Ctrl, control.

Next, the effects of inhibiting Pygo2 expression on the cell survival in human lung cancer cells were studied. Two weeks following Pygo2 shRNA or non-silencing control shRNA transfection and subsequent G418 selection, stable transfectants of A549 and H1299 cells were established. MTS proliferation (Fig. 3A) and colony formation (Fig. 3B) assays showed that knockdown of Pygo2 expression in the stable lines led to significant proliferative suppression when compared with the controls (for the two Pygo2 shRNAs: MTS, P<0.005; colony formation, P<0.001).

### shRNA knockdown of Pygo2 suppresses the growth of lung cancer in vivo

**Figure 4:**
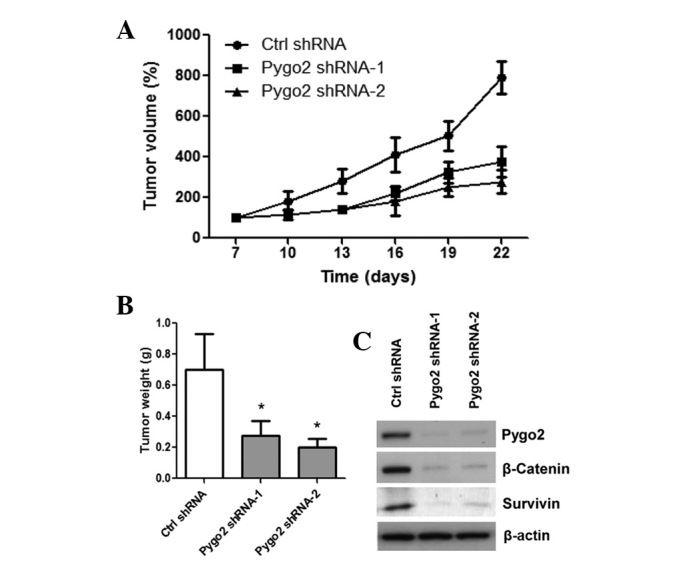
Pygo2 shRNAs suppress lung cancer growth *in vivo*. (A) Tumor size was monitored and measured every three days following inoculation of A549 cells stably transfected with control or Pygo2 shRNA. (B) Weight of the tumors measured at completion of the experiment. Data are presented as the mean ± SD (error bars). (C) Western blot analysis of resected tumors at completion of the experiment. Pygo2, Pygopus-2; shRNA, short hairpin RNA; Ctrl, control.

Finally, xenograft mouse models with A549 cells stably transfected with control or Pygo2 shRNAs were established. The stable lines were implanted into female BALB/c nude mice. Then, tumor formation was monitored and tumor mass was measured every three days. A significant reduction was observed in tumor size and mass of the Pygo2 shRNA tumors (n=5 for Pygo2 shRNA-1 and -2 groups) compared with those of the control shRNA tumors (n=5) (Fig. 4A; P=0.005). Following four weeks of tumor growth, mice were sacrificed and the tumors were collected for weight measurement. Tumor weight of the two Pygo2 shRNA treated groups was significantly less than that of the control shRNA treated group (Fig. 4B; P<0.001). In addition, the resected xenograft tumor specimens were examined by western blot analysis following completion of the *in vivo* experiment (Fig. 4C). Downregulation of Pygo2, cytosolic β-catenin and survivin proteins was observed in shRNA treated tumors compared with those of the control shRNA tumors, which was consistent with the *in vitro* results. Overall, the results suggested that Pygo2 may be a therapeutic target for lung cancer.

## Discussion

Aberrant activation of the canonical Wnt signaling pathway has been demonstrated in numerous types of cancer, including lung cancer (3,16). Several upstream components of the Wnt pathway have been previously reported to be dysregulated in lung cancer; for example, Wnt-1 and -2 were found to be upregulated in NCSLC cell lines and primary tissues. Inhibition of Wnt-1 or -2 by small interfering RNA or monoclonal antibodies was found to induce apoptosis in NSCLC cell lines (6). On the other hand, downregulation of Wnt-7a has been demonstrated in the majority of NSCLC cell lines and primary tissues, suggesting that it may act as a novel tumor suppressor in lung cancer. In addition, coexpression of Wnt-7a and Fzd-9 was found to inhibit NSCLC cell growth, indicating a ligand-receptor role for these proteins (8). Dvl, functioning downstream of the Fzd receptors as the mediator of Wnt signaling, has been previously reported to be overexpressed in 75% of NSCLC tissues. Inhibition of Dvl3 resulted in decreased TCF-dependent transcription and cell growth (9). In addition, epigenetic silencing of the Wnt antagonists has been found to be important for aberrant activation of the canonical Wnt pathway in lung cancer (10–12). Previous studies have also demonstrated the involvement of several Fzd receptors in various types of cancer (17–23). It has also been suggested that Pygo family members may be involved in β-catenin/TCF driven transcription in colorectal and breast cancer cells (5,13). The involvement of Pygo proteins in human lung cancer, however, remains largely unknown.

In the present study, the expression of Pygo2 in human lung cancer was examined. In addition, it was investigated whether Pygo2 expression correlates with β-catenin expression and is associated with aberrant Wnt pathway activation and cell proliferation in lung cancer. A significant overexpression of Pygo2 was demonstrated in primary lung cancer tissue samples when compared with their adjacent normal tissues, as well as in the examined lung cancer cell lines. Pygo2 and β-catenin proteins were also correlatively expressed and colocalized in the nuclei of lung cancer cell lines. These observations suggested that Pygo2 may be an important positive downstream effector of the canonical Wnt cascade in human lung cancer. To test the possible functional significance of the Pygo2 overexpression and coexpression with β-catenin in lung cancer, shRNA and stable transfection methods were used to knockdown endogenous Pygo2 expression in two lung cancer cell lines expressing the gene. Knocking down Pygo2 expression in these cells not only inhibited proliferation *in vitro* (demonstrated by MTS and colony formation assays), but also suppressed tumor growth *in vivo*. Knocking down Pygo2 expression was also accompanied by inhibition of the β-catenin/TCF-dependent transcriptional activity and, in turn, the canonical Wnt signaling in these cells, as demonstrated by a decrease in the levels of cytosolic β-catenin and its downstream target genes (such as cyclin D1 and survivin). These results indicated that Pygo2 may be important in aberrant activation of the canonical Wnt pathway that is critical for the proliferation and survival of lung cancer cells. Overall, the results of the present study suggested that Pygo2 may be a good target for the development of therapeutics to treat lung cancer. A small molecule or short peptide strategy blocking interaction between Pygo2 and β-catenin may be preferable in order to generate significant biological activity with minimal toxicity for the treatment of lung cancer. Several previous studies (24–26) have described strategies of developing potent inhibitors of Wnt signaling using synthetic peptides mimicking the β-catenin-binding domain on Bcl-9 protein and blocking the Bcl-9-β-catenin interaction. This peptide inhibitor is able to inhibit tumor growth in xenograft models, suggesting a potential therapeutic agent by targeting the transcriptional complex downstream of the canonical Wnt signaling in colon cancer. Such targeted strategies and agents may also benefit lung cancer patients demonstrating a Pygo2 tumor signature in the future. In conclusion, we propose that Pygo2 is a putative promising therapeutic target for human lung cancer. The observations of the current study may aid the development of more effective new agents targeting the Pygo2-mediated signaling pathway for this fatal disease.
